# Histopathological evaluation of non-melanoma skin cancer

**DOI:** 10.1186/1477-7819-12-159

**Published:** 2014-05-21

**Authors:** Ali Koyuncuer

**Affiliations:** 1 Department of Pathology, Antakya State Hospital, Antakya, Hatay, Turkey

**Keywords:** Histopathology, Non-melanoma skin cancer, Dermatopathology

## Abstract

**Background:**

Non-melanoma skin cancers (NMSCs) are the most frequently seen cancers worldwide.

**Methods:**

The medical records of patients diagnosed with basal cell carcinoma (BCC) and squamous cell carcinoma (SCC) in Hatay Antakya pathology laboratory between January 2010 and September 2012 were retrospectively included in the study. Tumors were categorized according to age, gender, anatomical localization, type, solitary-multiplicity, tumor diameter (0 to 2 mm, 2.1 to 6 mm and >6.1 mm), and presence of ulceration (BCCs), and morphological subtype, histopatological features and grades (SCCs).

**Results:**

A total of 136 tumors in 127 NMSC cases were examined. Solitary tumors were seen in 118 (92.9%), and multiple tumors in 9 (7.1%) patients. Mean age of the patients was 68.5 ± 13 years. BCC was observed in 96 (75.6%) and SCC in 31 (24.4%) patients. Mean diameter of all types of solitary and multiple tumors was 7.42 ± 3.49 mm. Nodular subtype focal cystic changes were observed in 49 (47.6%) patients. All tumors (solitary and multiple) were seen on the face (67.6%), scalp (11.8%), and ear (11%). Well differentiated SCCs were detected in 20 cases (64.5%); ulceration was observed in 58.1% of all tumors.

**Conclusions:**

Epidemiologic and histopathological investigations, routine skin scanning performed on the elderly population and dermatological examination will help to improve efficient health applications.

## Background

Non-melanoma skin cancers (NMSCs) are frequent in the United States with an estimated incidence of approximately two million cases per year [1]. NMSC caused 75,000 deaths between 1976 and 2000 [2]. NMSCs are mostly seen on the head or neck, and ≥90% of all skin cancers consist of basal cell carcinoma (BCC) or squamous cell carcinoma (SCC) [3]. Genetic factors, ultraviolet radiation (UVR), especially ultraviolet B (UVB) which contributes to the formation of SCCs and BCCs, are also implicated in the development of NMSCs [4]. In Hatay, Turkey, with a population of 1,474,000 people, where 32% of the city population live in the center of the city and the others in rural areas (latitude: 36 25′ 49″ N, longitude: 36 10′ 27″ E), most of the people work in agriculture, animal husbandry and commerce. In summer the climate is hot and arid, and in winters it is mild and rainy (characteristic Mediterranean climate). Average temperatures are; 20.2, 23.6, 23.3°C in June, July and August, and highest temperatures are 37.0, 40.8, and 40.4°C, respectively.

The aim of the present study was to clarify the clinicopathologic features and morphological characteristics of NMSCs diagnosed in skin biopsies in Hatay, where ultraviolet exposure might be higher than in other regions of Turkey.

## Methods

### Study design

This study was approved by the local Institutional Review Board (Antakya State Hospital). In the study, we re-examined 4 μm sections of formalin-fixed and paraffin-embedded basal cell and squamous cell skin cancer specimens, and stained them with hematoxylin and eosin, except for malignant melanoma, precancerous malignancies, non-invasive lesions and other malignancies in Hatay Antakya State Hospital’s pathology laboratory between January 2010 and September 2012. Tumors were categorized according to age, gender, anatomical localization, type, solitary/multiplicity, tumor diameter (0 to 2 mm, 2.1 to 6 mm and >6.1 mm), presence of ulceration (BCCs), and morphological subtype, histopatological features, and grades (SCCs). BCCs were categorized as nodular (solid), micronodular, cystic, superficial (superficial multifocal), pigmented, adenoid, infiltrating, sclerosing/morpheoform, keratocystic, basosquamous and adnexal differentiation, and mixed patterns. Other rarely seen types mentioned in the literature were not observed [3,4]. SCCs were categorized as grade 1 (well differentiated tumors), 2 (moderately differentiated tumors), 3 (poorly differentiated tumors), and 4 (anaplastic or undifferentiated tumors), based on the classification of the College of American Pathologists (CAP) and the TNM Classification of Malignant Tumours (International Union Against Cancer 2009) [5]. Patient age was categorized as 20 to 40 years, 41 to 50 years, 51 to 60 years, 61 to 70 years, 71 to 80 years, and >81 years. NMSCs were seen on the face (frontal, temporal, orbital, zygomatic, nasal, infraorbital, parotid, oral, buccal, mental regions), scalp, ear, neck, trunk, and extremities [6].

### Statistical analyses

Data were analyzed using the Statistical Package for Social Sciences (SPSS) software (version 15.0 for Windows; SPSS Inc., Chicago, IL, USA). The *t*-test was used for comparing averages, the chi-test was used for comparing dispersion of two groups, and the Kruskal-Wallis-H test was used for comparing dispersion of multi-groups. All differences associated with a chance probability of 0.05 or less were considered statistically significant. Continuous variables are presented as mean ± SD.

## Results

A total of 127 NMSC cases examined between January 2010 and September 2012 were reported.

### Solitary/multiple

Solitary tumors were seen in 118 (92.9%), and multiple tumors in 9 (7.1%) patients.

### Age

Mean age of the patients was 68.5 ± 13.6 (range 27 to 94) years. Mean ages of the cases diagnosed as BCC and SCC were 67.2 ± 13.9 and 72.4 ± 11.8 years, respectively. No statistically significant difference was found between groups (*P* = 0.062). The median age detected in men for BCC was 67.3 years, and in women was 67 years; for SCC, median ages were 69.8 years for men, and 76.7 years for women. Distribution of cases among age groups were as follows: 20 to 40 years, 2.4%; 41 to 50 years, 11.8%; 51 to 60 years, 11.8%; 61 to 70 years, 18.1%; 71 to 80 years, 37.8%; and >81 years, 18.1%. Median age of the cases with multiple tumors was 71.1 years. No statistically significant difference was found between age groups and cancer types (*P* = 0.410).

### Gender

Among 72 men (56.7% of the total) and 55 women (43.3% of the total), BCC was observed in 53 men (41.7%) and 43 women (33.8%). SCCs were detected in 19 men (15.06%) and 12 women (9.44%). Male or female dominancy was not observed among cases with multiple tumors. No statistically significant difference was found between genders and cancer types (*P* = 0.556).

### Cancer type

BCC was observed in 96 (75.6%) and SCC in 31 (24.4%) patients. Most (77.8%) of the multiple tumors were determined as BCC. Patient demographic features such as cancer type, gender and age are summarized in Table 1.

**Table 1:** Relation between cancer type, gender, age and rates in patients

**Cancer type**	**Male**		**Female**		**Total number**
	**Mean age (years)**	**n**	**Mean age (years)**	**n**	**n (%)**
BCC	67.3	53	67	43	96 (75.6)
SCC	69.8	19	76.7	12	31 (24.4)
Total		72*		55*	127 (100)

*Number (n). BCC, basal cell carcinoma; SCC, squamous cell carcinoma.

### Greatest diameter of the tumors

Mean diameter of all types of solitary and multiple tumors was 7.42 ± 3.49 mm. Mean tumor diameters for BCC and SCC were 7.08 ± 2.940 (range 2 to 15) mm and 8.48 ± 3.93 (range 3 to 22) mm, respectively. Mean diameters of all solitary and multiple tumors were 7.6 ± 3.311 mm and 7 ± 2.5 mm, respectively. A correlation was detected between cancer types and tumor dimensions (*P* = 0.030). Tumors were categorized according to their greatest diameters as: 0 to 2 mm (1.5%), 2.1 to 6 mm (45.6%), and ≥6.1 mm (52.9%).

### Basal cell carcinoma morphological subtype

Solitary and multiple tumors (total n = 103) in 96 BCC patients were evaluated. Nodular (solid) subtype focal cystic changes were observed in 49 (47.6%), mixed patterns in 29 (28.2%), infiltrating type in 13 (12.6%), adenoid in 7 (6.8%), micronodular in 3 (2.9%), superficial cancer in 1 and basosquamous in 1, respectively. Distribution of morphological subtypes of basal cell carcinoma is shown in Table 2.

**Table 2:** Basal cell carcinoma morphological subtypes distribution

**BCC morphological subtypes**	**n (%)**
Nodular (solid)	49 (47.6%)
Mixed patterns	29 (28.2%)
Infiltrating	13 (12.6%)
Adenoid	7 (6.8%)
Micronodular	3 (2.9%)
Superficial (multifocal)	1 (1.0%)
Basosquamous	1 (1.0%)
Total	103 (100.0%)

BCC, basal cell carcinoma.

Predominantly nodular subtype was detected among mixed types. Nodular (solid) types (pure or mixed) were the most frequently seen types of tumors (67%). Focal pigmentations were detected in 12.5% of all 12 BCC patients. No statistically significant difference was found between pigmentations and BCC subtypes (*P* = 0.927).

### Squamous cell carcinoma differentiation

Well-differentiated (grade 1) SCCs were detected in 20 cases (64.5%), and moderately differentiated (grade 2) SCCs were found in 35.5% of the cases. All multiple SCCs were moderately differentiated. In our study, grade 3 (poorly differentiated) and 4 (anaplastic or undifferentiated) tumors were not detected.

### Anatomic localization

**Figure 1:**
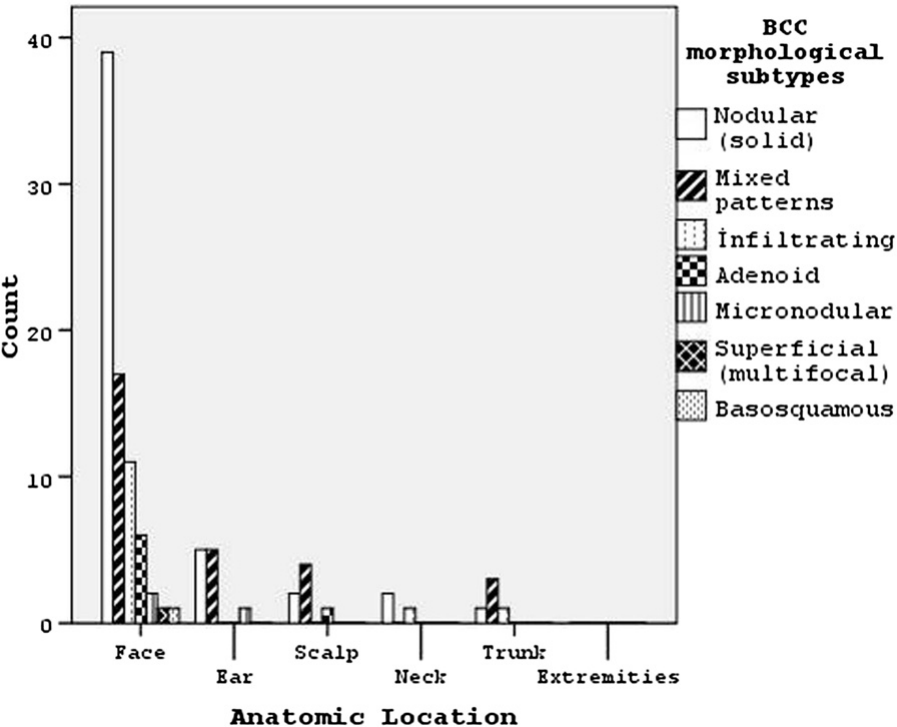
Morphological subtypes of basal cell carcinomas (BCCs) and their anatomic locations.

**Figure 2:**
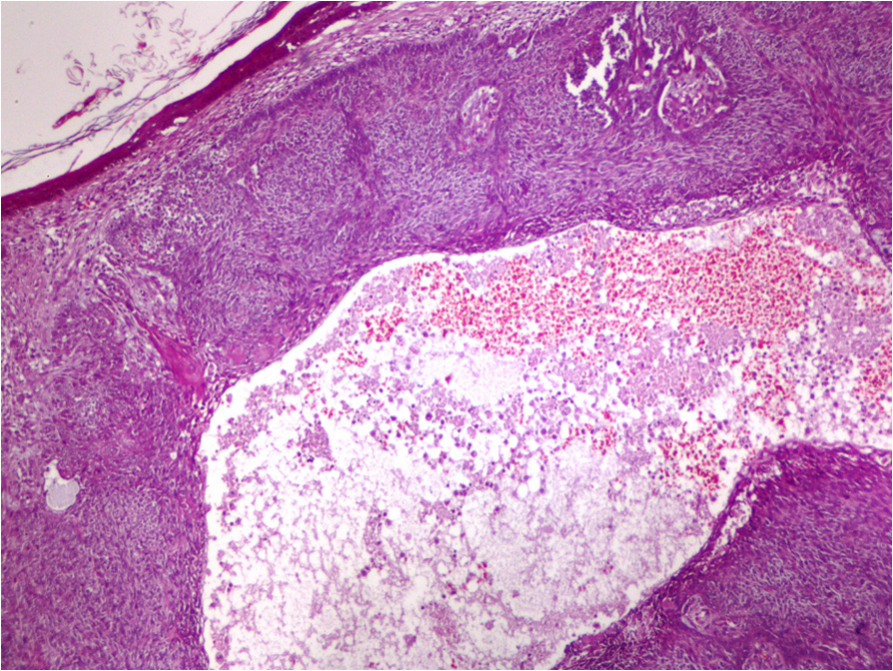
**Basal cell carcinoma of the nodular (solid) type, with cystic change present.** (Hematoxylin and eosin, x200).

**Figure 3:**
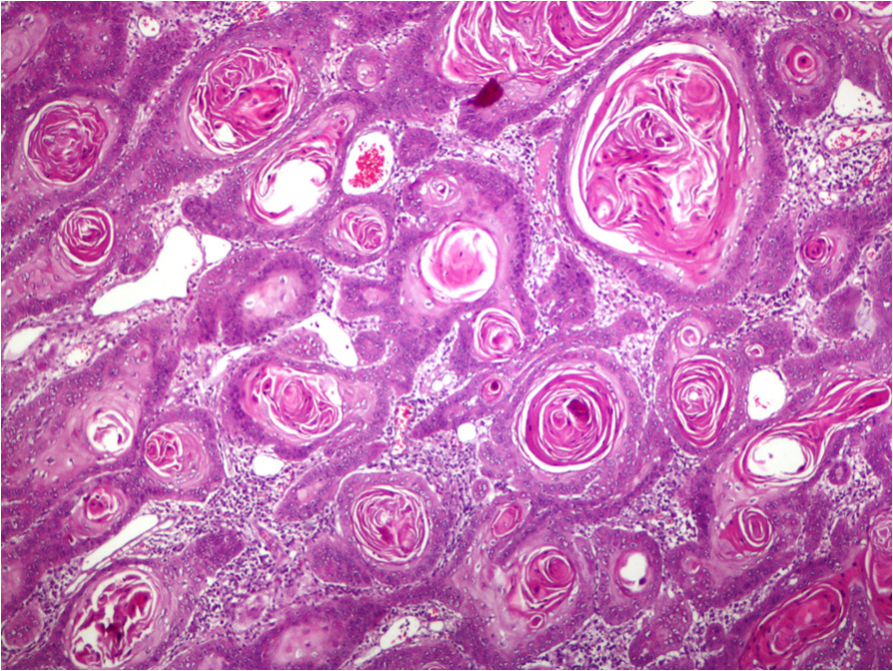
**Squamous cell carcinoma, well differentiated, shows keratocysts.** (Hematoxylin and eosin, x200).

All tumors (solitary and multiple) were seen on the face (67.6%), scalp (11.8%), and ear (11%). Significant differences were detected between cancer type and anatomic localization (*P* = 0.008), and 74.8% of BCCs were localized on the face. In our study, 92.7% of all BCCs (89 patients) were solitary, while 7 patients had BCCs on more than one anatomic location. BCCs were detected on the nose (n = 30; 29.1%), ear (10.7%), scalp (6.8%), and frontal region (5.8%). SCCs were seen on the face (n = 15; 8%) and scalp (n = 8). All multiple tumors were seen on the face, except for one patient (nose,4; zygomatic/buccal region, 4; mental region, 2; orbital region, 1; lip, 1; scalp, 1; ear, 1). SCCs were multiple in two patients (face, 1; ear, 1; scalp, 1; extremity, 1). When relations between BCC subtypes and anatomic localizations were investigated, nodular subtypes were seen on the face (76.9%; n = 39), nose (34.7%), frontal (10.2%) and orbital regions (10.2%). Figure 1 shows morphological subtypes of BCCs and their anatomic locations.Nodular (solid) types, consisting of a nodular nest of basal cell types which have peripheral palisading of lesional cell nuclei, a specialized stroma, tumor necrosis and focal cystic changes, were seen. In certain cases, particular nodular subtypes with one or more than one cystic space of different size and shape or cystic degenerations were observed. Nodular (solid) types, with cystic changes in the central part of the tumor nest, are shown in Figure 2. Mixed subtypes were seen on the face (58.6%), nose (64.7%), and ear (17%). Adenoid subtypes were seen on the face except in one patient. Adenoid type was characterized as nodular (solid) type, with mucoid appearance in tubular stroma, and gland-like structures (pseudoglandular appearance). Infiltrative subtypes were seen on the face (84.6%), and nose (45.5%). Histological features consisted of cord-like, elongated strands of basaloid cells infiltrating between collagen bundles, and palisading of the peripheral cells. Some cases, especially with nodular subtypes, had melanophages in the stroma and melanocytes within tumor islands which had melanin granules in dark-staining cytoplasm showed pigmentations. Along with mixed types in six patients and in patients with pure superficial multifocal types, on epidermal surface, ulcerations, and from epidermis to dermis buds and irregular proliferations, tumors which generate clefting between dermal layers, superficial type that had pigmentation on its center and melanin pigments on solid tumor nests were observed. Nodular subtypes was observed in all multiple BCCs (nodular; pure type, 6; mixed type, 1; adenoid,1; mixed, 1; micronodular, 1). SCCs were detected on the face (48.4%), scalp (26%), nose and extremities with decreasing order of frequencies. Well differentiated SCCs were detected on the face (40%), scalp (30%), and extremities (15%). Well differentiated SCC (grade 1) is a tumor which consists of irregular masses of epidermal cells which show decreased dermal proliferation. Abundant keratinization/keratocysts, abundant eosinophilic cytoplasm, nucleus that shows large vesicles, minimal pleomorphism, and minimal mitosis in the center of the tumor were seen (Figure 3). Moderately differentiated SCCs were seen on the face (63.6%). Moderately differentiated (grade 2) squamous cell carcinoma, nuclear and cytoplasmic pleomorphisms, keratin formation, horn cyst, and individually scattered keratinized cells were seen predominantly.

### Ulceration

Ulceration was observed in 58.1% of all tumors. Ulceration was detected in 55.3% of BCCs and 66.7% of SCCs. No statistically significant difference was found between ulceration and cancer types (*P* = 0.247). In cases with and without ulcerations, greatest diameters of the tumors were found to be 6.30 ± 3.438 mm and 8.23 ± 2.864 mm, respectively. A statistically significant inverse correlation was found between ulceration and the greatest diameter (*P* = 0.001).

## Discussion

NMSCs are malignant neoplasms that displays basal cell and squamuos cell differentiation [7]. Defined by Unna in 1896, solar radiation is the main etiologic factor for human NMSCs [8]. In 1934, Roffo demonstrated the effect of solar light on skin malignancies [9]. Exposure to sunlight is the most frequent principal factor in the etiology of squamous cells cancers. Along with ionized radiation, other risk factors for SCCs are chemical carcinogens, arsenic, immunosuppression, ulcers, and precursor lesions [10]. BCCs are the most frequently seen skin cancers and malignant neoplasia is mostly observed on the skins of Caucasians [11,12]. In our study, BCC was the most frequently seen NMSC.

BCC usually develops from solar radiation to the skin. Having fair skin, being employed outside, x-ray exposure, chickenpox scars, tattoos, and hair transplantation scars are other etiological factors [11]. The earth’s surface is exposed to 95% ultraviolet A (UVA, 320–400 mm), and 1 to 5% UVB (290–320 mm). UVB radiation may be more of a risk for BCC, and UVB causes BCC more commonly than UVA [8]. They include prolonged exposure to solar radiation is on the basis of risk for SCC. High ultraviolet exposure causes SCC more commonly than it causes BCC [7]. Smokers were asked about the type of tobacco product they used (cigarettes, cigars, pipe or a combination of these products). Smoking-related cancers are usually of the lung, lip, pharynx, larynx, salivary glands and leukemia, but Sitas and colleagues found a weak correlation between smoking and BCC [13]. McBride and colleagues [14] found that smoking did not increase the risk of SCC. De Hertog and colleagues [15] detected that smoking is an unrelated cause for skin SCC, reporting that there was no correlation between smoking and BCC and melanoma. Karagas and colleagues emphasized that the higher risk of SCC related with smoking value anymore training [16].

NMSCs have been shown to be BCCs in 80% of instances, and 20% are SCCs [17]. Our BCC and SCC rates were observed to be close to these percentages. A study in Australia showed that NMSCs were seen at an incidence of 884/100,000 population for BCC, and 387/100,000 for SCC [18]. Somewhat less than 5% of NMSCs are diagnosed before the age of 40 [19]. In our study their incidence was 97.6% which is in accordance with the literature. BCC is seen more frequently in older age groups. SCC occurs most frequently in elderly people. Incidence of SCCs increases from the age of 75 years [4,10].

In our study, BCC was observed more often in men. In the literature, age, gender and localization studies exist for both tumors. A study showed that BCCs were seen approximately at the age of 65 years [20], while another study demonstrated that BCCs were seen in women at a rate of 53.8% at a median age of 70.3 years [21]. The authors also recorded that SCCs were seen in men with an incidence of 50.8%, and at a mean age of 74.4 (±12.4) years [21]. In our study, SCCs were seen in men more than women and mean age of their onset was closer to those mentioned in the literature. Most (82.8%) of BCCs involved head and neck regions, and 17.2% of them were found on the trunk, and limb region. Andrade and colleagues [21] and Yap [22] showed that most BCCs were identified on the face (62.9%) (nose, 29.4%; zygomatic prominence, 21.4%, and forehead, 14.7%).

SCCs are frequently reported to be located on the head and neck area, with 51.9% on the face (zygomatic prominence, 31%; lower lip, 17.8%; ear, 11.7%; forehead, 11.5%, and nose, 10.1%), while the other most common site was the trunk [10,21,23]. In our study, BCC and SCC were seen most frequently on the face and neck area. In addition, SCC was more frequently seen on the face, and then on the scalp. A study showed that ulceration was not observed in 18% of cases with BCC [22]. In our study, ulceration was seen in more than half of the cases, and thus our rates were different from the literature findings. According to CAP, ulceration was not one of the high risk histologic features for SCC [5]. Cherpelis and colleagues noted that ulceration was not associated with the spread of the tumor [24]. BCC tumors of the neck and head had high relapse rates. While recurrences were not observed for lesions which were smaller than 2 mm, recurrence rates observed for 6 to 10 mm lesions and those greater than 30 mm in diameter were 8.8% and 23.1%, respectively [25]. Brantsch and colleagues [26] reported that tumors ≤2 mm did not show recurrence, those greater than 2.0 mm in thickness carried a risk of metastasis, and tumors bigger than 6 mm had higher risks of metastasis and local recurrence. Greatest diameter of the tumors was found to be ≥6.1 mm in 52.9% of the patients included in the study (72 cases), but any evidence of metastasis and death was not found in hospital and pathology records.

Various opinions have been suggested for the morphological subtypes of BCC. Weedon [3] and Haws and colleagues [27] opined that the mixed type is most frequently seen, but a study showed that the solid variant is most frequently seen [28], and superficial multicentric and solid-adenoid variants were observed. Puizina-Ivić and colleagues observed pure and mixed variants at a rate of 83.1% and 16.9%, respectively [28]. Nakjang and Kullavanijaya [29] opined that pigmentation was most frequently seen in solid, superficial and adenoid subtypes. Yap [22] suggested that the most frequently seen was the nodular type (95.3%) followed by superficial BCC; however, Scrivener and colleagues [20] reported that nodular, superficial, and morphoeiform types were seen at rates of 78.7%, 15.1% and 6.2%, respectively. In our study, the nodular subtype was seen at a rate in accordance with the literature, but mixed subtypes were ranked second. Corrêa and colleagues recorded that well differentiated types were the most frequently seen type of SCC, followed by the moderately differentiated type [30]. In our study, most of the SCCs were of the well differentiated type, and poorly differentiated (grade 3) and anaplastic or undifferentiated (grade 4) types were not observed. This was attributed to the development of lesions on visible areas of the body and early diagnosis.

## Conclusions

At the end of our study period in which age of onset, gender of the patients, histological type, subtype and anatomic locations of the NMSCs were examined, it is possible to say that exposure to ultraviolet radiation is the major potential etiologic factor for cancer considering the geographic and climatic factors of the Hatay region and that people are outdoor workers employed in agriculture and animal husbandry. Epidemiologic and histopathologic investigations, routine skin screening performed on the elderly population, and dermatological examination will help to improve efficient health applications. NMSCs were found in the those older than 60 years and most frequently on the face and, most often, the nodular subtype of BCC was detected.

## Abbreviations

BCC: basal cell carcinoma; CAP: College of American Pathologists; NMSC: non-melanoma skin cancers; SCC: squamous cell carcinoma; UVA: ultraviolet A; UVB: ultraviolet B; UVR: ultraviolet radiation.

## Competing interests

The author declares that he has no competing interests.
